# 724 Palliative Care Consultation in an Urban Burn Center

**DOI:** 10.1093/jbcr/irae036.267

**Published:** 2024-04-17

**Authors:** Miranda Haslam, Jeffrey Anderson

**Affiliations:** Temple University Hospital, Philadelphia, PA; Temple University Hospital, Philadelphia, PA

## Abstract

**Introduction:**

There is growing evidence supporting early palliative care consultation in many surgical patient populations; however, current literature regarding palliative care consultation in the burn population is limited. Given the significant morbidity and potential mortality associated with burn injuries, especially large total body surface area (TBSA) burn injuries, there is likely benefit to increased utilization of specialty palliative care in the burn patient population. In this study, we investigated current practice patterns of palliative care consultation at our burn center.

**Methods:**

A retrospective chart review was performed at our American Burn Association verified burn center of patients admitted from 2020 to 2023. Inclusion criteria included patients who presented with greater than or equal to 30% TBSA burns or experienced in-hospital mortality. Comparison was made between patients who did and did not receive palliative care consultation during their hospitalization. Student’s T-Test and Chi-Square Test were used for categorical and continuous variables, respectively.

**Results:**

A total of 38 patients met our inclusion criteria. 13 of the 38 patients (34%) received a palliative care consult during their admission, with an average of 10.2 days between admission and consultation. There was a trend towards older age and higher TBSA in patients who received a palliative care consultation (52.9 years vs. 49.2 years, 53.4% TBSA vs. 50.4% TBSA), though these differences were not statistically significant (p = 0.56, 0.33). There was no significant difference in likelihood of palliative care consultation based on presentation characteristics of intubation or inhalation injury. There was also no significant difference identified between patients who did and did not receive palliative care consultation in transition to comfort directed care, in-hospital death, or length of stay. In a subgroup analysis of patients who survived hospitalization, rates of home discharge were higher in those patients who did not receive palliative care consultation (p= 0.005).

**Conclusions:**

Opportunities exist for increased integration of specialty palliative care in the burn patient population. This study represents one of the first reviews of palliative care consultation utilization in an urban burn center patient population. Understanding of current practice patterns relating to palliative care consultation is an essential first step in developing practices to increase utilization of palliative care in the burn patient population.

**Applicability of Research to Practice:**

Significant opportunities exist for improved integration of specialty palliative care in the burn patient population. This research will be used to inform development of triggers for palliative care consultation at our burn center.

